# Association of metabolic syndrome with the incidence of hearing loss: A national population-based study

**DOI:** 10.1371/journal.pone.0220370

**Published:** 2019-07-26

**Authors:** Da Jung Jung, Kyung Do Han, Yang-Sun Cho, Chae Seo Rhee, Kyu-Yup Lee

**Affiliations:** 1 Department of Otorhinolaryngology-Head and Neck surgery, School of Medicine, Kyungpook National University, Kyungpook National University Hospital, Daegu, Korea; 2 Department of Biostatistics, College of Medicine, the Catholic University of Korea, Seoul, Korea; 3 Department of Otorhinolaryngology-Head and Neck Surgery, Samsung Medical Center, Sunkyunkwan University School of Medicine, Seoul, Korea; 4 Department of Otorhinolaryngology, Seoul National University College of Medicine, Seoul, Korea; University of Chicago, UNITED STATES

## Abstract

**Background & aims:**

Sensorineural hearing loss (HL) is one of the most common public health problems, and its prevalence increases with increasing life expectancy. An association between HL and metabolic syndrome (MetS) is suspected. Although previous epidemiological studies have investigated the association between the two variables, there have been conflicting conclusions. Therefore, we aimed to evaluate the association between the presence of MetS—and individual components of MetS—and HL, using a longitudinal design and a large-scale population.

**Methods:**

A total of 17,513,555 individuals who underwent national health screening between January 2009 and December 2010 were identified. Subject data from the Korean Health Insurance Review and Assessment Service were reviewed. A total of 11,457,931 subjects were ultimately included in the analysis. Baseline comorbidities were defined according to the ICD-10 code from the Korean Health Insurance Review and Assessment Service data. If the participants had an ICD-10 code for HL during the follow-up, they were defined as having incident HL. Criteria for MetS adhered to the revised National Cholesterol Education Program Adult Treatment Panel III.

**Results:**

There were 7,574,432 subjects without MetS and 3,883,499 with MetS. The incidence of HL in subjects without MetS and with MetS was 1.3% and 1.8% at 1 year, 4.1% and 5.2% at 3 years, and 6.8% and 8.6% at 5 years, respectively (*P* < 0.001). However, multivariate analyses revealed a negative association. Analyses according to the components of MetS demonstrated a positive association for those associated with dyslipidemia; however, the others exhibited an inverse association with HL. We also performed analyses using 4 groups according to the presence of MetS and the components of dyslipidemia. Univariate analysis revealed a positive association between the presence of MetS and HL; however, multivariate analysis revealed a positive association between the presence of dyslipidemia components and HL, regardless of the presence of MetS.

**Conclusion:**

Among the components of MetS, the association between low HDL or high TG levels and HL was most apparent. It is useful to evaluate each MetS component in isolation, such as the presence of low HDL or high TG levels, rather than the presence of MetS as a cluster of components.

## Introduction

Sensorineural hearing loss (HL) is one of the most common public health problems, and its prevalence increases with increasing life expectancy. Results from the Global Burden of Disease Study revealed that the prevalence of HL increased from 14.33% in 1990 to 18.06% in 2015 [1]. In addition, the study reported that HL was ranked fifth highest for disease years lived with disability in both developed and developing countries [2]. Age, excessive noise, and ear diseases are established risk factors for HL [3]. However, recent studies have demonstrated the association of HL with various cardiovascular and/or metabolic disorders that would cause injury to the nerves or vessels within the cochlea [4]. The World Health Organization has reported increasing trends in aging populations and the prevalence of various cardiovascular and/or metabolic diseases, which are projected to be associated with further increases in the prevalence of HL [3].

Metabolic syndrome (MetS), also known as syndrome X, is the term attributed to a cluster of a clustering of 5 medical conditions including the following: large waist circumference (WC); high blood pressure (BP); high fasting blood glucose (FBG); high triglyceride (TG) levels; and low levels of high-density lipoprotein (HDL) [5]. Individuals with ≥ 3 of the 5 components are usually diagnosed with MetS. The presence of MetS is closely associated with cardiovascular and/or metabolic diseases including diabetes mellitus (DM), hypertension (HTN), coronary artery disease, and stroke.

Many researchers have suggested that there are significant associations between cardio-metabolic disturbances and HL or MetS. Accordingly, an association between HL and MetS is also suspected. Although previous epidemiological studies have investigated the association between the two variables, there have been conflicting conclusions [6–12]. Most studies investigating the association, however, have been cross-sectional in design. Therefore, we aimed to evaluate the association between the presence of MetS—and individual components of MetS—and HL, using a longitudinal design and a large-scale population.

## Materials and methods

### Subjects

**Fig 1:**
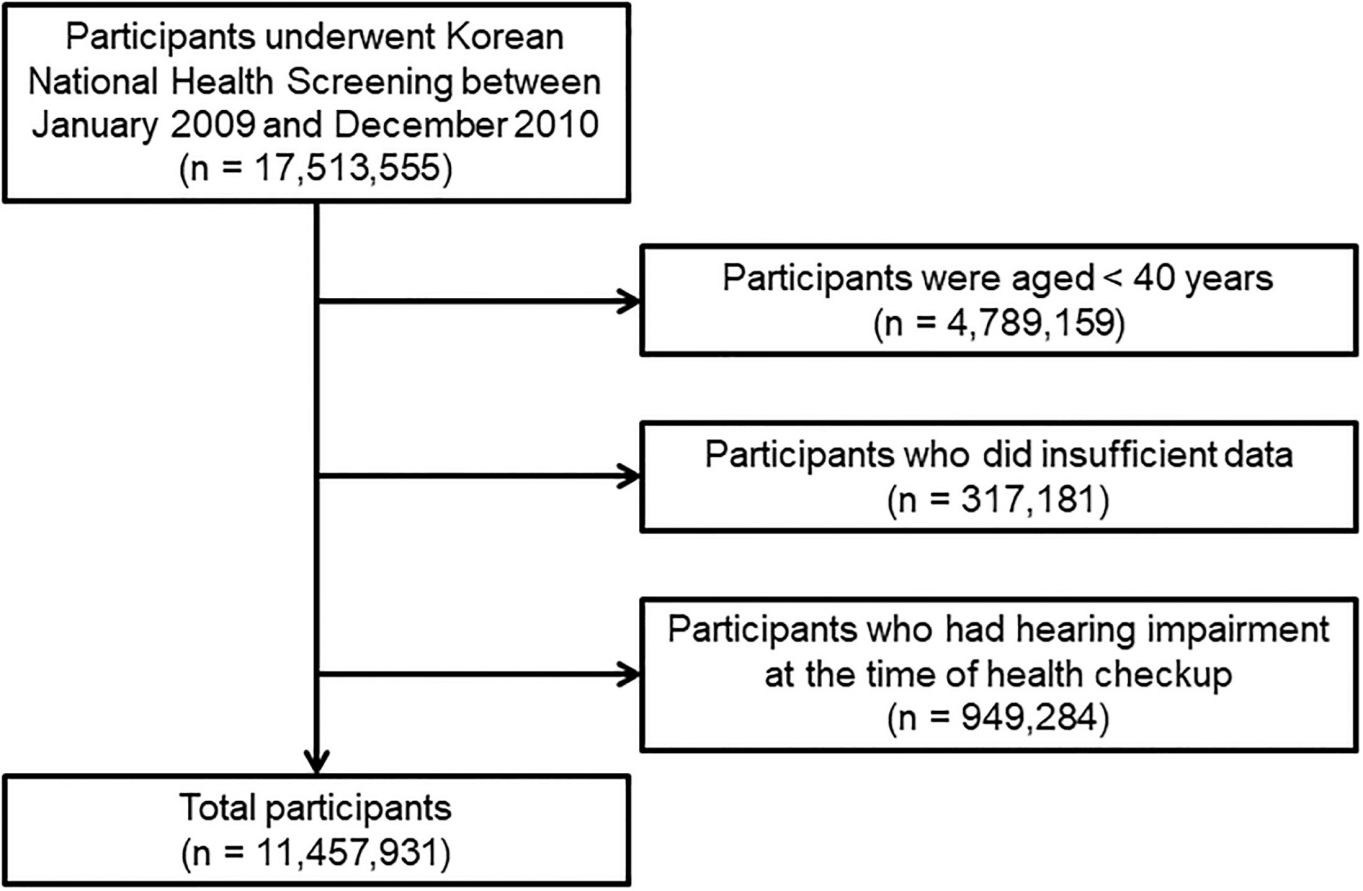
Flow chart.

A total of 17,513,555 individuals who underwent national health screening between January 2009 and December 2010 were identified (Fig 1). Subject data from the Korean Health Insurance Review and Assessment Service (KHIRAS) were reviewed. Our study involved the analysis of two databases, namely national health screening records collected between January 2009 and December 2010 and KHIRAS collected between January 2008 and December 2016. We first identified the baseline characteristics of the participants, including the presence or absence of MetS, MetS components, and comorbidities, from the national health screening data. Therefore, the presence or absence of MetS was evaluated at baseline alone. This database did not include the data regarding ICD-10 code, hearing thresholds, or questionnaires for HL. We also subsequently merged the data with KHIRAS. KHIRAS did not include the data for hearing thresholds, but included all ICD-10 codes accompanied with the dates when the relevant diagnosis was made during the follow-up period. Comorbidities were identified by reviewing International Classification of Diseases, Tenth Revision (ICD-10) codes during the previous year before the time of health screening. Among all subjects, those < 40 years of age (n = 4,789,159), with insufficient data (n = 317,181), or an ICD-10 code for HL during the previous year, before or at the time of health screening, were excluded. A total of 11,457,931 subjects were ultimately included in the analysis. This study was not a controlled study and the frequency of follow-up was not defined. In our study, the next follow-up point of the participants was defined as hospital visit point for admission or outpatient department. The follow-up interval or length of follow-up was variable. However, the last follow-up date could be identified. The study was approved by the Institutional Review Board (IRB) of Kyungpook National University Hospital (Daegu, South Korea; IRB No 2017-11-014-001). All personal identifiers were deleted prior to analysis. Therefore, the board waived the need for informed consent. The study was conducted in accordance with the principles that have their origin in the Declaration of Helsinki.

### Definitions

Age, sex, smoking habits, alcohol intake, exercise, presence of ear disease, annual income, body mass index (BMI), estimated glomerular filtration rate (eGFR), FBG level (mg/dL), systolic BP (mmHg), diastolic BP (mmHg), total cholesterol (mg/dL), and HDL (mg/dL), low-density lipoprotein (mg/dL), serum creatinine (mg/dL) and TG levels (mg/dL), were collected at health screening. Smoking habit was classified as non-smoker, ex-smoker, or current-smoker. Alcoholic intake was classified as non-alcohol, social alcohol consumption (< 30g per day), or heavy alcohol consumption (≥ 30g per day). Exercise was defined as strenuous exercise performed more than once per week. The presence of ear disease was defined as ICD-10 codes H60-62.8, H65-75, H80-83, and H90.0–90.2. Low income was defined as an annual income in the lower 20%. BMI was calculated using body weight divided by height squared (i.e., kg/m^2^). eGFR was calculated using the equation from the Modification of Diet in Renal Disease study: eGFR = 186 × serum creatinine − 1.154 × age − 0.203.

Baseline comorbidities were defined according to the ICD-10 code from the Korean Health Insurance Review and Assessment Service data. We defined DM as having FBG level ≥ 126 mg/dL or ICD-10 code E11-14; HTN as systolic BP ≥ 140 mmHg or diastolic BP ≥ 90 mmHg or ICD-10 code I10-13, I15. Further, cerebrovascular accident and heart disease were defined based on medical history; chronic kidney disease (CKD) was defined as eGFR *<* 60 ml/min/1.73 m^2^.

In Korea, the ICD-10 code is usually identified by a medical doctor. Therefore, HL was defined using ICD-10 code for sensorineural HL. HL was defined as ICD-10 codes H90.3–8, H91.1, H91.8–9 and, if the subject had HL during year before health screening, the subject was excluded from analysis. Subjects were followed from health screening to December 2016. If the participants had an ICD-10 code for HL during the follow-up, they were defined as having incident HL.

Criteria for MetS adhered to the revised National Cholesterol Education Program Adult Treatment Panel III [5]. Five components for MetS were defined as follows: WC, > 90 cm men and >80 cm women; FBG ≥ 100 mg/dL or a diagnosis of DM; BP ≥130/85 mmHg or a diagnosis of HTN; TG ≥150 mg/dL; and HDL < 40 mg/dL for men and < 50 mg/dL for women. MetS was considered to be present subjects with ≥ 3 of the 5 components of MetS.

### Statistical analysis

The data were analyzed using SAS version 9.4 (SAS, Cary, NC, USA). Categorical data were expressed as number (percentage), and continuous data were expressed as mean ± standard deviation. The distributions of continuous variables were evaluated according to the Kolmogorov-Smirnov criteria. Because the distribution of TG values was skewed, log-transformed values were used in this study. Distributions of categorical data were analyzed using the chi-squared test, and the mean values of continuous data were compared using the Student’s *t*-test. The incidence of HL was compared using Kaplan-Meier curve and Cox regression analyses; *P* <0.05 was considered to be statistically significant. Model 1 was adjusted for age and sex; model 2 was adjusted for age, sex, smoking habits, alcohol intake, exercise, and low income; model 3 was adjusted for age, sex, smoking habits, alcohol intake, exercise, low income, and BMI; and, finally, model 4 was adjusted for age, sex, smoking habits, alcohol intake, exercise, low income, BMI, and the presence of ear disease.

## Results

### Baseline characteristics

There were 7,574,432 subjects without MetS and 3,883,499 with MetS. The mean age of the subjects without MetS was 52.3 ± 10.0 years and 58.1 ± 10.6 years for those without MetS (Table 1). Follow-up periods for participants without MetS and those with MetS were 6.5 ± 1.4 and 6.4 ± 1.6 years, respectively. The prevalence of comorbidities, including DM, HTN, dyslipidemia, cerebrovascular accident, heart disease, CKD, or ear disease was greater in those with MetS than without MetS. Cardio-metabolic parameters, including BMI, eGFR, FBG, SBP, DBP, total cholesterol, HDL-C, LDL, and log(TG), were greater in those with MetS than in those without MetS.

**Table 1:** Baseline characteristics of participants.

Variables	Participants without MetS (n = 7,574,432)	Participants with MetS (n = 3,883,499)	*P*-value*
Age (years)	52.3 ± 10.0	58.1 ± 10.6	<0.001
Sex (men, %)	3,685,624 (48.7%)	1,866,383 (48.1%)	<0.001
Follow-up duration (years)	6.5 ± 1.4	6.4 ± 1.6	<0.001
Diabetes mellitus (%)	328,142 (4.3%)	1,019,125 (26.2%)	<0.001
Hypertension (%)	1,442,577(19.1%)	2,401,414 (61.8%)	<0.001
Dyslipidemia (%)	864,390 (11.4%)	1,833,986 (47.2%)	<0.001
Cerebrovascular accident (%)	66,024(1.5%)	81,653 (2.8%)	<0.001
Heart disease (%)	106,877 (2.3%)	198,715 (6.7%)	<0.001
Chronic kidney disease (%)	389,544 (5.1%)	423,047 (10.9%)	<0.001
Smoking habitus			<0.001
Non-smoker	4,880,623 (64.4%)	2,493,968 (64.2%)	
Ex-smoker	1,114,453 (14.7%)	628,780 (16.2%)	
Current smoker	1,579,356 (20.9%)	760,751 (19.6%)	
Alcohol habitus			<0.001
Abstinence	4,382,427 (57.9%)	2,403,013 (61.9%)	
Mild intake	2,757,928 (36.4%)	1,199,519 (30.9%)	
Heavy intake	434,077 (5.7%)	280,967 (7.2%)	
Exercise (%)	3,857,953 (50.9%)	1,814,147 (46.7%)	<0.001
Low income (%)	1,935,850 (25.6%)	981,273 (25.3%)	<0.001
Ear disease (%)	2,771,146 (36.6%)	1,623,511 (41.8%)	<0.001
Body mass index (kg/m^2^)	23.1 ± 2.7	25.6 ± 3.0	<0.001
eGFR (ml/min per 1.73m^2^)	87.3 ± 35.2	83.2 ± 33.6	<0.001
Fasting blood glucose (mg/dL)	94.2 ± 17.8	111.3 ± 31.8	<0.001
Waist circumference (cm)	78.4 ± 7.8	86.3 ± 7.9	<0.001
Systolic blood pressure (mmHg)	120.4 ± 14.4	131.3 ± 15.0	<0.001
Diastolic blood pressure (mmHg)	75.1 ± 9.7	80.8 ± 10.0	<0.001
Total cholesterol (mg/dL)	196.5 ± 34.2	204.1 ± 41.8	<0.001
High-density lipoprotein cholesterol (mg/dL)	57.4 ± 17.2	49.8 ± 17.4	<0.001
Low-density lipoprotein cholesterol (mg/dL)	117.0 ± 31.6	117.6 ± 39.1	<0.001
Log(Triglycerides)	0.96 ± 0.76	0.98 ± 0.73	<0.001

The data are expressed as counts (percentages) for categorical variables and as mean ± standard deviations for continuous variables.

*P values were determined using Student *t*-test for continuous variables and Pearson’s χ^2^ test for categorical variables.

Abbreviation: MetS, metabolic syndrome; eGFR, estimated glomerular filtration rate.

### Association between MetS and HL

**Fig 2:**
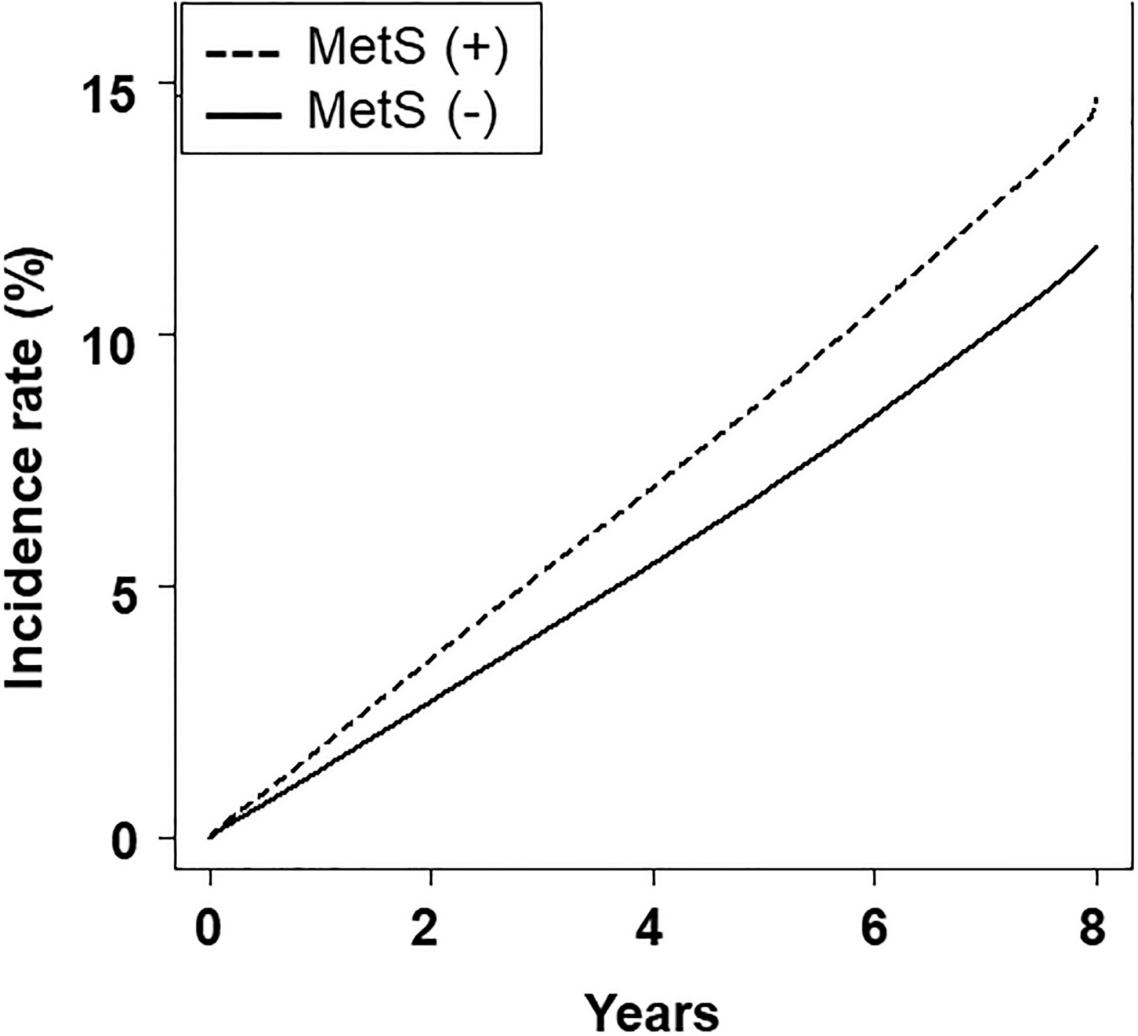
Kaplan-Meier curve according to the presence of metabolic syndrome.

The incidence of HL according the presence of MetS is shown in Fig 2. The incidence of HL in subjects without MetS and with MetS was 1.3% and 1.8% at 1 year, 4.1% and 5.2% at 3 years, and 6.8% and 8.6% at 5 years, respectively (*P* < 0.001). Hazard ratios for HL in MetS in models 1, 2, 3, and 4 were 1.014 (95% confidence interval [CI] 1.010−1.018), 1.016 (95% CI 1.012−1.020), 1.019 (95% CI 1.015−1.023), and 1.001 (95% CI 0.997−1.005), respectively (*P* < 0.001 for model 1, 2 and 3, and *P* = 0.567 for model 4). Hazard ratios for HL according to the number of MetS components are shown in Table 2. The trend demonstrated statistical significance; however, model 4 revealed that participants with 1, 2, or 3 components of MetS had an inverse association for incident HL compared with those without components of MetS.

**Table 2:** Cox-regression analyses of hearing loss according to numbers of metabolic syndrome components.

Variables	Model 1	Model 2	Model 3	Model 4
0 (ref)				
1	0.986 (0.980−0.992)	0.990 (0.984−0.995)	0.991 (0.985−0.997)	0.991 (0.985−0997)
2	0.983 (0.977−0.989)	0.989 (0.983−0.995)	0.992 (0.986−0.998)	0.988 (0.982−0.994)
3	0.994 (0.987−1.000)	1.001 (0.994−1.007)	1.005 (0.998−1.011)	0.992 (0.985−0.999)
4	1.008 (1.001−1.015)	1.013 (1.006−1.020)	1.018 (1.011−1.026)	0.994 (0.986−1.001)
5	1.013 (1.004−1.022)	1.019 (1.010−1.027)	1.025 (1.016−1.035)	0.991 (0.982−1.001)

The data are expressed as hazard ratio (95% confidence interval). Model 1 was adjusted for age and sex; model 2 was adjusted for age, sex, smoking habitus, alcohol habitus, exercise, and low income; model 3 was adjusted for age, sex, smoking habitus, alcohol habitus, exercise, low income, and body mass index; and model 4 was adjusted for age, sex, smoking habitus, alcohol habitus, exercise, low income, body mass index, and presence of ear disease. For models 1, 2, and 3, the *P* values for trends were < 0.001, while for model 4, the *P* value for trends was 0.0086.

The associations between each MetS component and HL are summarized in Table 3. Subjects with WC, BP or FBG demonstrated a lower risk for HL compared with those without these components; however, those with TG or HDL had a higher risk for HL compared with those without these components.

**Table 3:** Cox-regression analyses of hearing loss according to presence of each metabolic syndrome component.

Variables	Model 1	Model 2	Model 3	Model 4
Waist circumference	0.991 (0.987−0.995)	0.991 (0.987−0.995)	0.986 (0.981−0.991)	0.980 (0.975−0.985)
Blood pressure	0.953 (0.949−0.956)	0.954 (0.950−0.957)	0.951 (0.947−0.955)	0.947 (0.943−0.951)
Fasting blood glucose	0.970 (0.967−0.974)	0.973 (0.970−0.977)	0.973 (0.969−0.976)	0.971 (0.968−0.975)
Triglycerides	1.027 (1.023−1.031)	1.036 (1.033−1.040)	1.038 (1.035−1.042)	1.022 (1.018−1.026)
High-density lipoprotein	1.083 (1.079−1.088)	1.079 (1.075−1.083)	1.082 (1.078−1.086)	1.059 (1.055−1.063)

The data are expressed as hazard ratio (95% confidence interval). Reference for each analysis was participants without each of the components. Model 1 was adjusted for age and sex; model 2 was adjusted for age, sex, smoking habitus, alcohol habitus, exercise, and low income; model 3 was adjusted for age, sex, smoking habitus, alcohol habitus, exercise, low income, and body mass index; and model 4 was adjusted for age, sex, smoking habitus, alcohol habitus, exercise, low income, body mass index, and presence of ear disease. All *P* values for trends were < 0.001.

### Subgroup analyses for identifying an association between MetS and HL

Subgroup analyses according to sex and age were performed. Model 4 revealed statistically significant results for men and women 40–64 years of age (S1 Table). There was an inverse association between the two variables in men, and a positive association in women 40–64 years of age. The association between the number of each MetS component and HL were analyzed (S2 Table). Model 4 revealed that men with 1, 2, 3, or 4 MetS components exhibited an inverse association with HL compared with men without any MetS components. A positive association was shown in the two variables in women 40–64 years of age, but not women ≥ 65 years of age. The presence of the large WC component was inversely associated with the risk for HL in men ≥ 65 years of age and women of all ages (S3 Table). The presence of a high BP or high FBG component was inversely associated with the risk for HL in men and women of all ages. The presence of high TG or low HDL component was positively associated with risk for HL in men and women of all ages.

Subgroup analyses were performed according to the presence of ear disease. For participants without ear disease, hazard ratios for HL of MetS in models 1, 2, and 3 were 0.998 (95% CI 0.992−1.003), 0.999 (95% CI 0.994−1.005), and 1.002 (95% CI 0.996−1.008), respectively (*P* values for models 1, 2, and 3 were 0.398, 0.777, and 0.582, respectively). For those with ear disease, hazard ratios for HL of MetS in models 1, 2, and 3 were 0.997 (95% CI 0.991−1.002), 0.999 (95% CI 0.994−1.004), and 1.004 (95% CI 0.999−1.010), respectively (*P* values for models 1, 2, and 3 were 0.188, 0.694, and 0.132, respectively). S4 and S5 Tables summarize the associations between the number of each MetS component or the presence of each MetS component and HL. The association between the number of MetS components or each MetS component and HL was similar for all participants.

### Association between dyslipidemia components and HL

Kaplan-Meier curve analysis revealed that the presence of MetS was associated with a high incidence of HL, regardless of the presence of high TG or low HDL levels (S1 Fig). For subjects with high HDL and low TG levels and without MetS, the incidence of HL was 1.3% at 1 year, 4.1% at 3 years, and 6.8% at 5 years. For subjects with low HDL or high TG levels and without MetS, the incidence of HL was 1.3% at 1 year, 4.1% at 3 years, and 6.8% at 5 years. For subjects with high HDL and low TG levels and with MetS, the incidence of HL was 1.7% at 1 year, 5.2% at 3 years, and 8.4% at 5 years. For subjects with low HDL or high TG levels and with MetS, HL incidence was 1.8% at 1 year, 5.3% at 3 years, and 8.6% at 5 years.

However, multivariate analysis revealed that subjects without MetS, and with high TG or low HDL levels, exhibited the highest risk for the incidence of HL (S6 Table). Those with MetS but without high TG and low HDL levels demonstrated the lowest risk for the incidence of HL. The presence of MetS had an inverse association with HL; however, the presence of high TG or low HDL levels demonstrated a stronger positive association with HL, regardless of the presence of MetS.

## Discussion

The present study was longitudinal investigation using a large-scale population aimed at identifying the association between MetS and HL. First, we analyzed the association between two variables using all participants, and the results revealed a negative association on multivariate analyses. Analyses according to the components of MetS demonstrated a positive association for those associated with dyslipidemia; however, the others exhibited an inverse association with HL. Secondarily, we performed subgroup analyses according to age and sex, and the presence of ear disease. There was positive association between the presence of MetS and HL in women aged 40–64 years, and an inverse association between the two variables in men all ages. Analyses according to MetS components yielded similar results for all subjects. We also performed analyses using 4 groups according to the presence of MetS and the components of dyslipidemia. Univariate analysis revealed a positive association between the presence of MetS and HL; however, multivariate analysis revealed a positive association between the presence of dyslipidemia components and HL, regardless of the presence of MetS.

Although some previous studies have reported a negative association between the presence of MetS and HL, most have demonstrated a positive association between the two variables. Most studies, however, were performed using a cross-sectional design. Some studies analyzed the association between MetS components and HL [7,9,11,12]. Han et al. reported a positive association between WC, HDL, or FBG and HL, while other studies reported a positive association with only FBG and positive association with HDL [7,9,12]. There were inconsistent results regarding the association between individual MetS components and HL. Our results demonstrated a negative association between the presence of MetS and HL. Two components of dyslipidemia among the 5 MetS components were associated with the incidence of HL, similar to the results reported by Sun et al. [7]. The other components were inversely associated with the incidence of HL.

WC, FBG, and BP were closely associated with DM or HTN, and epidemiological studies investigating the association between DM or HTN and HL have reported inconsistent results [13–16]. The inverse association found in our study may be consistent with these investigations. We suggest that individuals with these problems need regular medical support compared with those without, and may take various medications, which may be protective or a hazard to HL. These may be associated with inverse or negative associations between WC, FBG or BP and the incidence of HL.

Some clinical studies have reported an association between dyslipidemia and HL [12,17]. Previous researches involving guinea pigs fed a lipid-rich diet reported showed vacuolar edema and degeneration of the stria vascularis as possible pathognomonic changes in dyslipidemia [18,19]. A decrease in nitric oxide production and an increase in reactive oxygen species levels caused by dyslipidemia can induce hearing impairment [20–22]. In addition, previous research has demonstrated that HDL has anti-inflammatory, anti-oxidant, and anti-apoptotic effects, which may attenuate the pathological changes induced by dyslipidemia [23,24]. These data suggest that dyslipidemia is associated with development of HL. Our study revealed no association between MetS—defined as a group—and HL, although a conflicting effect may be associated with a hazard effect for dyslipidemia and a protective effect for the other components.

Nevertheless, our study had some inherent drawbacks that should be addressed, the first of which was its retrospective design. Second, co-morbidities, such as DM, HTN, heart disease, and cerebrovascular accident, were defined using ICD-10 codes or initially collected on simple histories or laboratory findings. We also did not collect data regarding hearing thresholds, and HL was simply defined as an ICD-10 code. Although ICD-10 code was determined by a medical doctor, the diagnosis could range widely from a simple patient complaint to a thorough evaluation for HL. In addition, we did not collect data regarding subject medications or drug classes. Third, our data did not include those regarding noise exposure, occupation, or history of ear disease. HL is associated with excessive noise exposure and/or history of ear diseases, which are important risk factors. However, we attempted to mitigate these problems by performing subgroup analyses or multivariate analyses including ear disease.

Although our study had some important limitations, it was based on a population that was virtually all ≥ 40 years of age. In addition, our study design was longitudinal with a median follow-up duration of 6.4–6.5 years, which may have also overcome these limitations. Further well-designed, prospective longitudinal studies are, however, needed to evaluate the association between the presence of MetS—or individual MetS components—and HL.

In conclusion, the results of our study suggest that MetS did not demonstrate a definite positive association with the incidence of HL compared with individuals without MetS. Among the components of MetS, the association between low HDL or high TG levels and HL was most apparent. It is useful to evaluate each MetS component in isolation, such as the presence of low HDL or high TG levels, rather than the presence of MetS as a cluster of components.

## Supporting information

S1 Fig Kaplan—Meier curve according to the presence of MetS and the presence of low HDL or high TG (*P* < 0.001). Abbreviation: MetS, participants with metabolic syndrome; Non-MetS, participants without metabolic syndrome; TG, high triglyceride level; HDL, low high-density lipoprotein level; Non-TG, low triglyceride level; Non-HDL; high high-density lipoprotein level.
(TIF)

S1 Table Cox-regression analyses of hearing loss according to the presence of metabolic syndrome. (DOC)

S2 Table Cox-regression analyses of hearing loss according to the number of metabolic syndrome components. (DOC)

S3 Table Cox-regression analyses of hearing loss according to the presence of each metabolic syndrome component. (DOC)

S4 Table Cox-regression analyses of hearing loss according to the number of metabolic syndrome components. (DOC)

S5 Table Cox-regression analyses of hearing loss according to the presence of each metabolic syndrome component. (DOC)

S6 Table Cox-regression analyses of hearing loss according to the presence of MetS and the presence of HDL or TG. (DOC)
