# Supplementary figures and images for: Association of metabolic syndrome with the incidence of hearing loss: A national population-based study
Source: PLoS One. 2019 Jul 26;14(7):e0220370. doi: 10.1371/journal.pone.0220370 (PMC6660075; doi:10.1371/journal.pone.0220370)

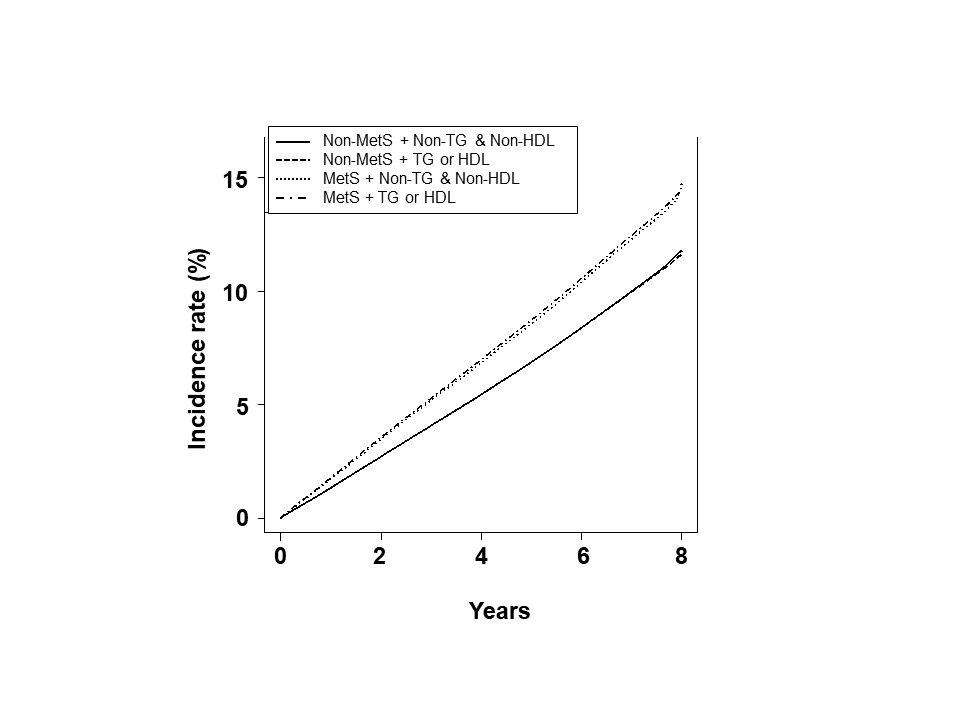

Supplement: S1 Fig — Abbreviation: MetS, participants with metabolic syndrome; Non-MetS, participants without metabolic syndrome; TG, high triglyceride level; HDL, low high-density lipoprotein level; Non-TG, low triglyceride level; Non-HDL; high high-density lipoprotein level. (TIF) [file pone.0220370.s001.tif]
