# Supplementary material for: Association of metabolic syndrome with the incidence of hearing loss: A national population-based study
Source: PLoS One. 2019 Jul 26;14(7):e0220370. doi: 10.1371/journal.pone.0220370 (PMC6660075; doi:10.1371/journal.pone.0220370)
Supplement: S1 Table — (DOC) [file pone.0220370.s002.doc]

| **Subgroup** | **Model 1** | | **Model 2** | | **Model 3** | | **Model 4** | |
| --- | --- | --- | --- | --- | --- | --- | --- | --- |
|  | HR (95% CI) | *P* | HR (95% CI) | *P* | HR (95% CI) | *P* | HR (95% CI) | *P* |
| Men, 40-64 aged | 0.998 (0.991−1.005) | 0.609 | 1.002 (0.995−1.009) | 0.624 | 1.000 (0.993−1.008) | 0.920 | 0.991 (0.984−0.998) | 0.016 |
| Men, ≥65 aged | 1.030 (1.020−1.040) | <0.001 | 1.020 (1.010−1.030) | <0.001 | 1.005 (0.995−1.016) | 0.339 | 0.986 (0.976−0.997) | 0.010 |
| Women, 40-64 aged | 1.017 (1.011−1.024) | <0.001 | 1.018 (1.011−1.024) | <0.001 | 1.037 (1.030−1.044) | <0.001 | 1.014 (1.006−1.021) | <0.001 |
| Women, ≥65 aged | 1.021 (1.012−1.030) | <0.001 | 1.020 (1.011−1.029) | <0.001 | 1.023 (1.014−1.033) | <0.001 | 1.001 (0.991−1.010) | 0.897 |

Model 1 was adjusted for age and sex; model 2 was adjusted for age, sex, smoking habitus, alcohol habitus, exercise, and low income; model 3 was adjusted for age, sex, smoking habitus, alcohol habitus, exercise, low income, and body mass index; and model 4 was adjusted for age, sex, smoking habitus, alcohol habitus, exercise, low income, body mass index, and presence of ear disease. Abbreviation: HR, hazard ratio; CI, confidence interval.
