# Supplementary material for: Association of metabolic syndrome with the incidence of hearing loss: A national population-based study
Source: PLoS One. 2019 Jul 26;14(7):e0220370. doi: 10.1371/journal.pone.0220370 (PMC6660075; doi:10.1371/journal.pone.0220370)
Supplement: S2 Table — (DOC) [file pone.0220370.s003.doc]

| **Variables** | **Model 1** | **Model 2** | **Model 3** | **Model 4** |
| --- | --- | --- | --- | --- |
| Men, 40-64 aged |  |  |  |  |
| 1 | 0.959 (0.949−0.969) | 0.964 (0.954−0.974) | 0.962 (0.953−0.972) | 0.965 (0.955−0.975) |
| 2 | 0.942 (0.932−0.952) | 0.951 (0.941−0.960) | 0.948 (0.938−0.958) | 0.951 (0.941−0.961) |
| 3 | 0.947 (0.937−0.957) | 0.957 (0.947−0.967) | 0.953 (0.942−0.964) | 0.951 (0.940−0.962) |
| 4 | 0.970 (0.958−0.982) | 0.977 (0.965−0.989) | 0.971 (0.959−0.984) | 0.960 (0.947−0.973) |
| 5 | 1.001 (0.983−1.019) | 1.005 (0.987−1.024) | 0.997 (0.978−1.017) | 0.975 (0.956−0.994) |
| Men, ≥65 aged |  |  |  |  |
| 1 | 0.968 (0.950−0.985) | 0.965 (0.948−0.983) | 0.959 (0.942−0.977) | 0.957 (0.939−0.974) |
| 2 | 0.958 (0.941−0.976) | 0.953 (0.936−0.971) | 0.940 (0.923−0.958) | 0.934 (0.917−0.951) |
| 3 | 0.987 (0.969−1.006) | 0.979 (0.961−0.997) | 0.958 (0.940−0.977) | 0.941 (0.923−0.959) |
| 4 | 0.994 (0.974−1.014) | 0.979 (0.960−0.999) | 0.954 (0.935−0.974) | 0.928 (0.909−0.948) |
| 5 | 1.048 (1.022−1.074) | 1.026 (1.000−1.052) | 0.988 (0.962−1.015) | 0.953 (0.928−0.979) |
| Women, 40-64 aged |  |  |  |  |
| 1 | 1.000 (0.992−1.009) | 1.001 (0.993−1.010) | 1.012 (1.003−1.020) | 1.010 (1.001−1.018) |
| 2 | 1.012 (1.003−1.021) | 1.013 (1.004−1.023) | 1.035 (1.025−1.045) | 1.025 (1.015−1.034) |
| 3 | 1.022 (1.012−1.032) | 1.023 (1.013−1.033) | 1.052 (1.041−1.063) | 1.030 (1.020−1.041) |
| 4 | 1.025 (1.014−1.037) | 1.026 (1.015−1.038) | 1.063 (1.050−1.076) | 1.028 (1.016−1.040) |
| 5 | 1.015 (1.000−1.030) | 1.016 (1.001−1.031) | 1.063 (1.047−1.080) | 1.020 (1.004−1.036) |
| Women, ≥65 aged |  |  |  |  |
| 1 | 0.989 (0.966−1.013) | 0.989 (0.966−1.013) | 0.991 (0.968−1.015) | 0.983 (0.960−1.006) |
| 2 | 0.993 (0.971−1.016) | 0.993 (0.971−1.015) | 0.997 (0.975−1.020) | 0.980 (0.958−1.003) |
| 3 | 1.003 (0.982−1.026) | 1.003 (0.981−1.025) | 1.009 (0.986−1.032) | 0.982 (0.960−1.004) |
| 4 | 1.020 (0.998−1.043) | 1.019 (0.996−1.041) | 1.026 (1.003−1.050) | 0.987 (0.964−1.010) |
| 5 | 1.020 (0.997−1.044) | 1.019 (0.995−1.043) | 1.028 (1.004−1.054) | 0.981 (0.958−1.006) |

The data are expressed as hazard ratio (95% confidence interval). Reference was participants without each of the metabolic syndrome components.

Model 1 was adjusted for age and sex; model 2 was adjusted for age, sex, smoking habitus, alcohol habitus, exercise, and low income; model 3 was adjusted for age, sex, smoking habitus, alcohol habitus, exercise, low income, and body mass index; and model 4 was adjusted for age, sex, smoking habitus, alcohol habitus, exercise, low income, body mass index, and presence of ear disease. For models 1, 2, and 3, all *P* values for trends were < 0.001. For model 4, *P* values for trends were < 0.001 for men all ages or women aged 40–64 years, and 0.591 for women aged ≥65 years.
