# Supplementary material for: Association of metabolic syndrome with the incidence of hearing loss: A national population-based study
Source: PLoS One. 2019 Jul 26;14(7):e0220370. doi: 10.1371/journal.pone.0220370 (PMC6660075; doi:10.1371/journal.pone.0220370)
Supplement: S3 Table — (DOC) [file pone.0220370.s004.doc]

| **Subgroup** | **Model 1** | | **Model 2** | | **Model 3** | | **Model 4** | |
| --- | --- | --- | --- | --- | --- | --- | --- | --- |
|  | HR (95% CI) | *P* | HR (95% CI) | *P* | HR (95% CI) | *P* | HR (95% CI) | *P* |
| Waist circumference |  |  |  |  |  |  |  |  |
| Men, 40-64 aged | 1.008 (1.000−1.015) | 0.044 | 1.003 (0.995−1.010) | 0.501 | 1.000 (0.991−1.010) | 0.928 | 0.996 (0.987−1.006) | 0.448 |
| Men, ≥65 aged | 1.022 (1.011−1.033) | <0.001 | 1.010 (1.000−1.021) | 0.059 | 0.976 (0.963−0.989) | <0.001 | 0.968 (0.955−0.981) | <0.001 |
| Women, 40-64 aged | 0.966 (0.959−0.973) | <0.001 | 0.968 (0.960−0.975) | <0.001 | 0.988 (0.979−0.997) | 0.011 | 0.983 (0.974−0.992) | <0.001 |
| Women, ≥65 aged | 0.990 (0.981−0.999) | 0.026 | 0.991 (0.982−1.000) | 0.045 | 0.988 (0.977−0.999) | 0.029 | 0.982 (0.972−0.993) | 0.002 |
| Blood pressure |  |  |  |  |  |  |  |  |
| Men, 40-64 aged | 0.938 (0.932**−**0.944) | <0.001 | 0.937 (0.931−0.943) | <0.001 | 0.933 (0.927**−**0.940) | <0.001 | 0.935 (0.929−0.941) | <0.001 |
| Men, ≥65 aged | 0.964 (0.954−0.975) | <0.001 | 0.958 (0.948−0.969) | <0.001 | 0.947 (0.937−0.958) | <0.001 | 0.942 (0.931−0.952) | <0.001 |
| Women, 40-64 aged | 0.956 (0.950−0.962) | <0.001 | 0.958 (0.952−0.964) | <0.001 | 0.964 (0.958−0.970) | <0.001 | 0.958 (0.952−0.964) | <0.001 |
| Women, ≥65 aged | 0.962 (0.953−0.972) | <0.001 | 0.961 (0.952−0.971) | <0.001 | 0.960 (0.950−0.970) | <0.001 | 0.948 (0.939−0.958) | <0.001 |
| Fasting blood glucose |  |  |  |  |  |  |  |  |
| Men, 40-64 aged | 0.955 (0.949−0.961) | <0.001 | 0.959 (0.952−0.965) | <0.001 | 0.957 (0.951−0.964) | <0.001 | 0.961 (0.955−0.968) | <0.001 |
| Men, ≥65 aged | 0.975 (0.966−0.984) | <0.001 | 0.971 (0.962−0.980) | <0.001 | 0.965 (0.956−0.974) | <0.001 | 0.962 (0.953−0.972) | <0.001 |
| Women, 40-64 aged | 0.972 (0.966−0.979) | <0.001 | 0.975 (0.968−0.981) | <0.001 | 0.980 (0.973−0.986) | <0.001 | 0.976 (0.970−0.983) | <0.001 |
| Women, ≥65 aged | 0.973 (0.965−0.982) | <0.001 | 0.974 (0.965−0.982) | <0.001 | 0.973 (0.965−0.982) | <0.001 | 0.967 (0.959−0.976) | <0.001 |
| Triglyceride |  |  |  |  |  |  |  |  |
| Men, 40-64 aged | 0.998 (0.991−1.004) | <0.001 | 1.014 (1.007−1.020) | <0.001 | 1.014 (1.007−1.020) | <0.001 | 1.007 (1.000**−**1.013) | 0.047 |
| Men, ≥65 aged | 1.028 (1.018−1.038) | <0.001 | 1.029 (1.019−1.040) | <0.001 | 1.021 (1.011−1.032) | <0.001 | 1.006 (0.996−1.016) | 0.279 |
| Women, 40-64 aged | 1.058 (1.051−1.065) | <0.001 | 1.059 (1.052−1.065) | <0.001 | 1.069 (1.062−1.076) | <0.001 | 1.045 (1.038−1.051) | <0.001 |
| Women, ≥65 aged | 1.050 (1.041−1.059) | <0.001 | 1.049 (1.040−1.058) | <0.001 | 1.051 (1.042−1.060) | <0.001 | 1.027 (1.018−1.036) | <0.001 |
| High-density lipoprotein |  |  |  |  |  |  |  |  |
| Men, 40-64 aged | 1.096 (1.088−1.104) | <0.001 | 1.091 (1.083−1.099) | <0.001 | 1.094 (1.086−1.102) | <0.001 | 1.070 (1.062−1.078) | <0.001 |
| Men, ≥65 aged | 1.087 (1.076−1.098) | <0.001 | 1.071 (1.060−1.082) | <0.001 | 1.065 (1.054−1.076) | <0.001 | 1.039 (1.028−1.050) | <0.001 |
| Women, 40-64 aged | 1.079 (1.072−1.085) | <0.001 | 1.075 (1.069−1.081) | <0.001 | 1.083 (1.076−1.089) | <0.001 | 1.060 (1.054−1.067) | <0.001 |
| Women, ≥65 aged | 1.060 (1.050−1.069) | <0.001 | 1.057 (1.048−1.066) | <0.001 | 1.058 (1.049−1.067) | <0.001 | 1.036 (1.027−1.045) | <0.001 |

Reference for each analysis was participants without each of the components. Model 1 was adjusted for age and sex; model 2 was adjusted for age, sex, smoking habitus, alcohol habitus, exercise, and low income; model 3 was adjusted for age, sex, smoking habitus, alcohol habitus, exercise, low income, and body mass index; and model 4 was adjusted for age, sex, smoking habitus, alcohol habitus, exercise, low income, body mass index, and presence of ear disease. Abbreviation: HR, hazard ratio; CI, confidence interval.
