# Supplementary material for: Association of metabolic syndrome with the incidence of hearing loss: A national population-based study
Source: PLoS One. 2019 Jul 26;14(7):e0220370. doi: 10.1371/journal.pone.0220370 (PMC6660075; doi:10.1371/journal.pone.0220370)
Supplement: S4 Table — (DOC) [file pone.0220370.s005.doc]

| **Variables** | **Model 1** | **Model 2** | **Model 3** |
| --- | --- | --- | --- |
| Participants without ear disease |  |  |  |
| 1 | 0.982 (0.974−0.990) | 0.985 (0.977−0.993) | 0.986 (0.977−0.994) |
| 2 | 0.970 (0.962−0.978) | 0.975 (0.967−0.984) | 0.976 (0.968−0.985) |
| 3 | 0.974 (0.966−0.983) | 0.98 (0.971−0.989) | 0.982 (0.972−0.991) |
| 4 | 0.985 (0.975−0.995) | 0.989 (0.979−0.999) | 0.99 (0.980−1.001) |
| 5 | 0.983 (0.970−0.996) | 0.986 (0.973−1.000) | 0.989 (0.975−1.003) |
| Participants with ear disease |  |  |  |
| 1 | 1.118 (1.108−1.127) | 0.992 (0.984−1.001) | 0.995 (0.986−1.003) |
| 2 | 1.223 (1.213−1.233) | 0.992 (0.984−1.000) | 0.996 (0.988−1.005) |
| 3 | 1.314 (1.303−1.325) | 0.993 (0.984−1.001) | 0.998 (0.989−1.007) |
| 4 | 1.404 (1.392−1.417) | 0.989 (0.980−0.999) | 0.994 (0.985−1.003) |
| 5 | 1.489 (1.473−1.505) | 0.986 (0.975−0.998) | 0.991 (0.980−1.003) |

The data are expressed as hazard ratio (95% confidence interval). Reference was participants without each of the metabolic syndrome components. Model 1 was adjusted for age and sex; model 2 was adjusted for age, sex, smoking habitus, alcohol habitus, exercise, and low income; and model 3 was adjusted for age, sex, smoking habitus, alcohol habitus, exercise, low income, and body mass index. For participants without ear disease, all *P* values for trends were < 0.001. For participants with ear disease, *P* values for models 1, 2, and 3 were 0.185, 0.621, and 0.589, respectively.
