# Supplementary material for: Association of metabolic syndrome with the incidence of hearing loss: A national population-based study
Source: PLoS One. 2019 Jul 26;14(7):e0220370. doi: 10.1371/journal.pone.0220370 (PMC6660075; doi:10.1371/journal.pone.0220370)
Supplement: S5 Table — (DOC) [file pone.0220370.s006.doc]

| **Variables** | **Model 1** | **Model 2** | **Model 3** |
| --- | --- | --- | --- |
| Participants without ear disease |  |  |  |
| Waist circumference | 0.983 (0.977−0.989) | 0.981 (0.975−0.987) | 0.977 (0.969−0.984) |
| Blood pressure | 0.943 (0.938−0.948) | 0.943 (0.938−0.948) | 0.941 (0.936−0.946) |
| Fasting blood glucose | 0.970 (0.965−0.976) | 0.972 (0.967−0.977) | 0.972 (0.967−0.978) |
| Triglycerides | 1.01 (1.004−1.015) | 1.019 (1.014−1.025) | 1.022 (1.016−1.027) |
| High-density lipoprotein | 1.065 (1.059−1.071) | 1.062 (1.056−1.068) | 1.065 (1.059−1.071) |
| Participants with ear disease |  |  |  |
| Waist circumference | 1.122 (1.116−1.129) | 0.980 (0.975−0.986) | 0.981 (0.976−0.987) |
| Blood pressure | 1.213 (1.207−1.219) | 0.954 (0.949−0.959) | 0.955 (0.950−0.960) |
| Fasting blood glucose | 1.082 (1.077−1.087) | 0.968 (0.963−0.973) | 0.971 (0.966−0.976) |
| Triglycerides | 1.132 (1.126−1.138) | 1.016 (1.010−1.021) | 1.022 (1.017−1.027) |
| High-density lipoprotein | 1.212 (1.206−1.218) | 1.054 (1.049−1.060) | 1.052 (1.047−1.058) |

The data are expressed as hazard ratio (95% confidence interval). Reference for each analysis was participants without each of the components. Model 1 was adjusted for age and sex; model 2 was adjusted for age, sex, smoking habitus, alcohol habitus, exercise, and low income; and model 3 was adjusted for age, sex, smoking habitus, alcohol habitus, exercise, low income, and body mass index. The *P* values for trends in all models were < 0.001.
