# Supplementary material for: Association of metabolic syndrome with the incidence of hearing loss: A national population-based study
Source: PLoS One. 2019 Jul 26;14(7):e0220370. doi: 10.1371/journal.pone.0220370 (PMC6660075; doi:10.1371/journal.pone.0220370)
Supplement: S6 Table — (DOC) [file pone.0220370.s007.doc]

| **Groups** | **Model 1** | **Model 2** | **Model 3** | **Model 4** |
| --- | --- | --- | --- | --- |
| Non-MetS combined with Non-TG & Non-HDL (ref) |  |  |  |  |
| Non-MetS combined TG or HDL | 1.023 (1.017−1.028) | 1.031 (1.026−1.037) | 1.032 (1.026−1.037) | 1.019 (1.014−1.024) |
| MetS combined with Non-TG & Non-HDL | 0.958 (0.948−0.968) | 0.958 (0.948−0.968) | 0.961 (0.951−0.971) | 0.959 (0.949−0.969) |
| MetS combined with TG or HDL | 1.026 (1.022−1.030) | 1.031 (1.027−1.036) | 1.034 (1.029−1.038) | 1.000 (0.996−1.004) |
| MetS combined with Non-TG & Non-HDL (ref) |  |  |  |  |
| Non-MetS combined with Non-TG & Non-HDL | 1.044 (1.033−1.055) | 1.044 (1.033−1.055) | 1.041 (1.030−1.052) | 1.045 (1.034−1.056) |
| Non-MetS combined TG or HDL | 1.068 (1.056−1.079) | 1.077 (1.065−1.088) | 1.073 (1.062−1.085) | 1.073 (1.061−1.085) |
| MetS combined with TG or HDL | 1.071 (1.060−1.082) | 1.077 (1.066−1.088) | 1.076 (1.064−1.087) | 1.059 (1.048−1.070) |
| MetS combined with TG or HDL (ref) |  |  |  |  |
| Non-MetS combined with Non-TG & Non-HDL | 0.975 (0.971−0.979) | 0.970 (0.966−0.974) | 0.967 (0.963−0.972) | 0.987 (0.983−0.992) |
| Non-MetS combined TG or HDL | 0.997 (0.991−1.002) | 1.000 (0.995−1.005) | 0.998 (0.993−1.004) | 1.013 (1.008−1.019) |
| MetS combined with Non-TG & Non-HDL | 0.933 (0.924−0.943) | 0.929 (0.919−0.938) | 0.930 (0.920−0.939) | 0.945 (0.935−0.954) |

The data are expressed as hazard ratio (95% confidence interval). Reference was Non-MetS combined with Non-TG & Non-HDL. Model 1 was adjusted for age and sex; model 2 was adjusted for age, sex, smoking habitus, alcohol habitus, exercise, and low income; and model 3 was adjusted for age, sex, smoking habitus, alcohol habitus, exercise, low income, and body mass index. All *P* values were < 0.001 except those for the analysis of Non-MetS combined with TG or HDL. Abbreviation: Non-MetS, participants without metabolic syndrome; Non-TG, low triglyceride level; Non-HDL, high high-density lipoprotein cholesterol level; TG, high triglyceride level; HDL, low high-density lipoprotein cholesterol level; MetS, metabolic syndrome.
